# Supplementary material for: The Influence of Public Health Faculty on College and University Plans During the COVID-19 Pandemic
Source: Front Public Health. 2022 Jan 14;9:745232. doi: 10.3389/fpubh.2021.745232 (PMC8795676; doi:10.3389/fpubh.2021.745232)
Supplement: Supplementary file 1 [file Data_Sheet_1.docx]

# Appendix and Supplemental Materials

Equation A1. Two-level Regression

Level 1: ${ln\left( \frac{P\left( Y_{i,j}\geq{Threshold}_{k} \right)}{P\left( Y_{i,j}<{Threshold}_{k} \right)} \right)}_{i,j}=\beta_{0,j}+\beta_{1}{X1}_{i,j}+\beta_{2}{X2}_{i,j}+\beta_{3}{X3}_{i,j}+\beta_{4}{X4}_{i,j}+\beta_{5,j}{X5}_{i,j}$

Level 2 $\beta_{0,j}=\gamma_{0,0}+u_{0,j}$

Level 2 $\beta_{5,j}=\gamma_{5,0}+u_{5,j}$

where *i* and *j* are index numbers that represent the *i^th^* college/university within each *j^th^* state

*k* is an index number that represents the threshold (cut point) the divides the ordinal scale into two (in total, five thresholds are used for the 6-level modality restriction scale: (1) Levels 2-6 vs. 1; (2) Levels 3-6 vs. 1-2; (3) Levels 4-6 vs. 1-3; (4) Levels 5-6 vs. 1-4; and (5) Level 6 vs. 1-5)

$\beta_{0,j}$ represents the intercept for the model for each *j^th^* state, which includes both a fixed component $\gamma_{0,0}$ (reported as the fixed intercept(s) in each model) and a component that varies by state (which is constrained to have an overall mean of 0; the variance is reported in the model results)

$\beta_{1}$ through $\beta_{4}$ represent the fixed slopes for the CEPH accreditation, enrollment, COVID-19 rate, and AAUP variables, respectively

$\beta_{5,j}$ represents the slope for the Governor’s Party variable, which includes both a fixed component $\gamma_{5,0}$ (reported as the fixed slope in each model) and a component that varies by state (which is constrained to have an overall mean of 0; the variance is reported in the model results)
